# Supplementary material for: AI-driven discovery of synergistic drug combinations against pancreatic cancer
Source: Nat Commun. 2025 Apr 29;16:4020. doi: 10.1038/s41467-025-56818-6 (PMC12041571; doi:10.1038/s41467-025-56818-6)
Supplement: Supplementary file 1 — Supplementary Information [file 41467_2025_56818_MOESM1_ESM.pdf]

Supplementary Information

AI-driven discovery of synergistic drug combinations against pancreatic cancer

Mohsen Pourmousa[1+], Sankalp Jain[1+], Elena Barnaeva[1], Wengong Jin[2], Joshua Hochuli[3], Zina Itkin[1], Travis Maxfield[3], Cleber Melo-Filho[3], Andrew Thieme[3], Kelli Wilson[1], Carleen Klumpp-Thomas[1], Sam Michael[1], Noel Southall[1], Tommi Jaakkola[2], Eugene N. Muratov[3,4], Regina Barzilay[2], Alexander Tropsha[3,4], Marc Ferrer[1], Alexey V. Zakharov[1*].

[1] National Center for Advancing Translational Sciences (NCATS), National Institutes of Health, 9800 Medical Center Drive, Rockville, Maryland 20850, United States.
[2] Computer Science and Artificial Intelligence Laboratory, Massachusetts Institute of Technology, Cambridge, MA 02139, USA.
[3] Laboratory for Molecular Modeling, Division of Chemical Biology and Medicinal Chemistry, UNC Eshelman School of Pharmacy, University of North Carolina, Chapel Hill, NC, 27599, USA.
[4] Predictive, LLC. Raleigh, NC, 27614, USA.

+ authors contribute equally

* Address for correspondence: 9800 Medical Center Dr, Rockville, Maryland 20850, USA; Telephone: 301-480-9847; E-mail: alexey.zakharov@nih.gov.

Supplementary Figures

Supplementary Fig. 1: **Reproducibility of combination experiments as measured by synergy metrics.**

a, Beta values of 496 drug combinations across two replicates show moderate correlation. **b,** ExcessHSA values for the same combinations demonstrate higher variability; the inset plot (excluding outliers) reveals improved correlation. Gamma values, which provide a more consistent measure of synergy, are presented in the main manuscript (Fig. 2). Source data are provided as a Source Data file.

Supplementary Fig. 2: **Random forest regression model predictions for synergy using Morgan 2048 and RDKit descriptors.**

a, Regression results using Morgan 2048 fingerprints as features show moderate correlation, with Pearson's coefficient indicated. **b,** RDKit descriptors yield comparable model performance. Each combination was evaluated using a one-compound-out cross-validation scheme. Source data are provided as a Source Data file.

Supplementary Fig. 3: **Classification model performance using Morgan 2048 and RDKit descriptors.**

a, ROC curves for Morgan 2048-based classification models. **b,** ROC curves for RDKit descriptor-based models. 32 ROC curves (not shown for clarity) have average AUC and standard deviation of 0.78 ± 0.09. The blue line represents the average ROC curve, with the gray shaded area showing ± 1 standard deviation. The red dashed line indicates random prediction. Gamma ≤ 0.95, synergistic; Gamma > 0.95, non-synergistic. Source data are provided as a Source Data file.

Supplementary Fig. 4: **ROC curve of the MIT graph convolutional network model.** Shown is the ROC curve for the first cross-validation fold of the graph convolutional network model developed by MIT. Gamma < 0.95 is considered synergistic, and Gamma ≥ 0.95 is non-synergistic. Source data are provided as a Source Data file.

Supplementary Fig. 5: **Distribution of predicted Gamma scores across all drug combinations.** Distribution of predicted gamma scores across all 1.5M+ drug combinations evaluated by the graph convolutional model. The gamma score, a measure of synergy, is plotted on the x-axis, with the frequency of combinations corresponding to each score shown on the y-axis. Source data are provided as a Source Data file.

Supplementary Fig. 6: **Top compounds and their associated average Gamma scores.** Frequency and average Gamma scores of the 10 most frequently occurring compounds in synergistic combinations. Each bar represents a compound, with the length of the bar indicating the frequency of combinations involving that compound. The color gradient along the bar corresponds to the compound's average gamma score, with the color bar on the right providing a reference for interpreting these scores. Source data are provided as a Source Data file.

Supplementary Fig. 7: **Validation of synergy predictions for experimentally tested combinations.**

Retrospective evaluation of synergy predictions for 88 combinations nominated by the final models from NCATS, UNC, and MIT. Predicted synergy scores are compared to experimental results. Source data are provided as a Source Data file.

Supplementary Fig. 8: **Network analysis of mechanisms of action (MoAs) in synergistic combinations.**

The network depicts MoA interactions identified in 307 synergistic combinations (Gamma < 0.95). Node sizes and edge widths are proportional to MoA and MoA–MoA frequency, respectively. Abbreviations in alphabetical order: 5-HT2A Antagonists, 5-HT2A; AKT Inhibitor, AKT; BTK Inhibitor, BTK; Bcl-xL Inhibitor, Bcl-xL; Brd4 Inhibitor, BRD4; CDK1/2/3/7/9 Inhibitor, CDK1,2,3,7,9; CDK1/2/5/9 Inhibitor, CDK1,2,5,9; CDK4/6 Inhibitor, CDK4,6; Cardiac glycoside, Cardi-glyc; Cyclooxygenase-2 Inhibitor, COX2; Dietary supplement & part of traditional chinese medicines; alkaloid known to have several pharmacological effects including anti-tumor activity; inducer of apoptosis, Diet-Supp; Dihydrofolate Reductase (DHFR) Inhibitors, DHFR; Farnesoid X Receptor Agonist, FXR; GSK-3 Inhibitor, GSK3; HDAC Inhibitor, HDAC; IAP Inhibitor, IAP; IDO Inhibitor, IDO; IKK beta Inhibitor, IKKβ; ITK inhibitor, ITK; Isocitrate dehydrogenase inhibitor, IDH; Jak/Tyk/Flt Inhibitor, JAK,TYK,FLT; MDM2 (hdm2) Inhibitor, MDM2; Mutant IDH1 Inhibitor, mut-IDH1; Mutant p53 Activator, mut-p53; NAMPT Inhibitor, NAMPT; NOS2 Expression Inhibitor, NOS2; Nicotinic acid, NicAcid; Nuclear export Inhibitor, NucExp; PARP Inhibitor, PARP; PI3K Inhibitor, PI3K; PKC Inhibitor, PKC; Phosphodiesterase III/Va (PDE) Inhibitor, PDE; Polo-like Kinase-1 (Plk-1) Inhibitor, PLK1; Proteasome Inhibitor, Proteasome; Protein kinase C theta Inhibitor, PRKCT; RIP1 Kinase Inhibitor, RIP1; Src Kinase Inhibitor, Src; Survivin Inhibitor, Survivin; TLR 7, 8, 9 Antagonist, TLR7,8,9; Tachykinin NK3

Antagonist, NK3R; Wee1 Kinase Inhibitor, Wee1; alpha2-Adrenoceptor Antagonist, α-Adrenoceptor; mTORC1/2 Inhibitor, mTORC1,2. Source data are provided as a Source Data file.

Supplementary Fig. 9: **Network analysis of MoAs in random combinations**. The analysis took the 64 unique compounds from the 307 synergistic combinations and generated 307 random combinations, repeating this 1,000 times. Node sizes and edge widths are proportional to MoA and MoA–MoA frequency, respectively. Each MoA is abbreviated for simplicity; for example, "HDAC" refers to "HDAC Inhibitor", where HDAC is a protein name. Supplementary Fig. 8 provides details of abbreviations. Source data are provided as a Source Data file.

Supplementary Fig. 10: **Matrix blocks from the NSC-319726 + AZD-8055 combination assay.** Normalized activity of combinations is represented as a heat map, with 0 corresponding to full cytotoxicity and 100 to no cytotoxicity. The color gradient from black to red illustrates the progression from minimal to maximal activity, with red indicating higher activity and black representing lower activity.

Supplementary Fig. 11: **Screening plate example with assay controls and combinations.** A screening plate example displaying responses to negative and positive controls, along with 12 combinations (highlighted by a yellow rectangle for one combination). Each 1536-well plate included DMSO as the negative control (IC0) and Bortezomib, a well-established cancer drug, as the positive control (IC100), located in the leftmost columns.

Supplementary Tables

Supplementary Table 1: Definitions of key synergy metrics in drug combination studies

Supplementary Table 2: Summary of NCATS machine learning results. AUC of ROC curve ± standard error of 32 values corresponding to 32 folds.

Supplementary Table 3: UNC modeling results

Supplementary Table 4: Cross-validation accuracy of the MIT graph convolutional network model on each of the five cross validation folds.

Supplementary Table 5: List of 26 strongly synergistic combinations with gamma < 0.5.

Supplementary Table 6. Small molecule screening data

Supplementary Figures

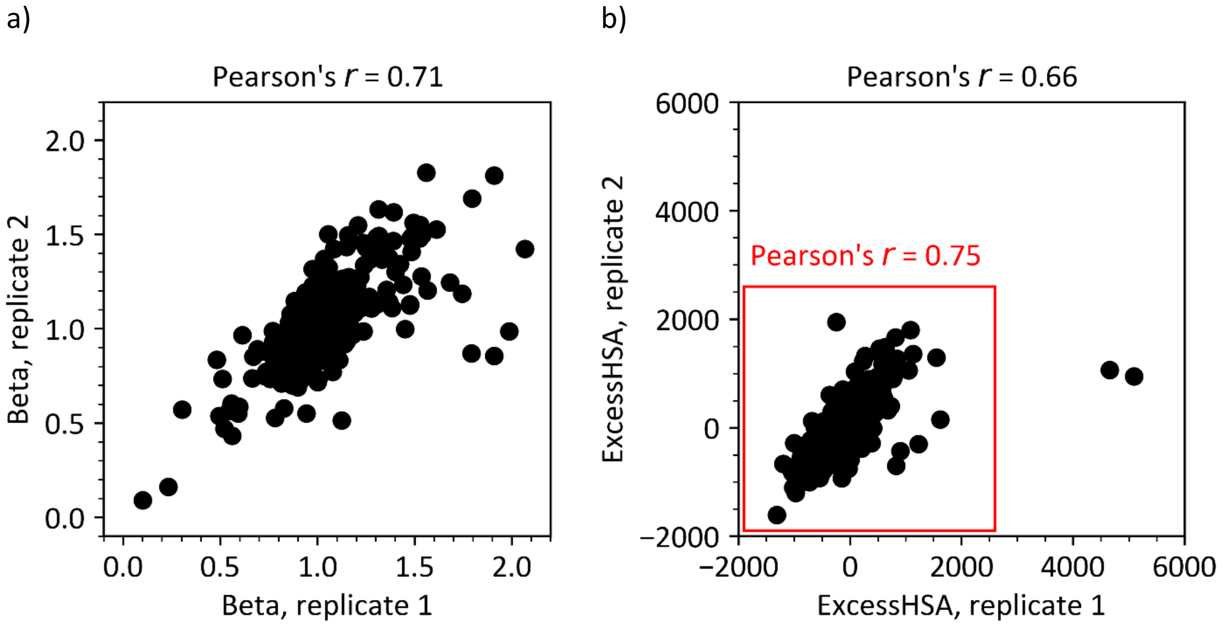

Supplementary Fig. 1: **Reproducibility of combination experiments as measured by synergy metrics.**
a, Beta values of 496 drug combinations across two replicates show moderate correlation. **b,** ExcessHSA values for the same combinations demonstrate higher variability; the inset plot (excluding outliers) reveals improved correlation. Gamma values, which provide a more consistent measure of synergy, are presented in the main manuscript (Fig. 2). Source data are provided as a Source Data file.

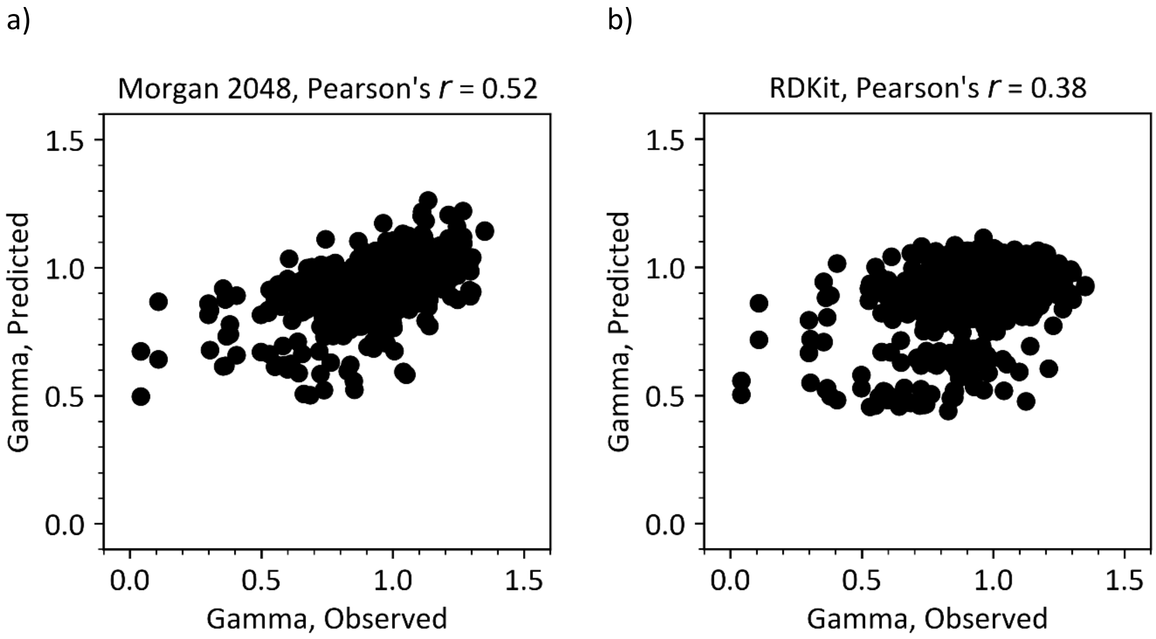

Supplementary Fig. 2: **Random forest regression model predictions for synergy using Morgan 2048 and RDKit descriptors.**

a, Regression results using Morgan 2048 fingerprints as features show moderate correlation, with Pearson's coefficient indicated. **b,** RDKit descriptors yield comparable model performance. Each combination was evaluated using a one-compound-out cross-validation scheme. Source data are provided as a Source Data file.

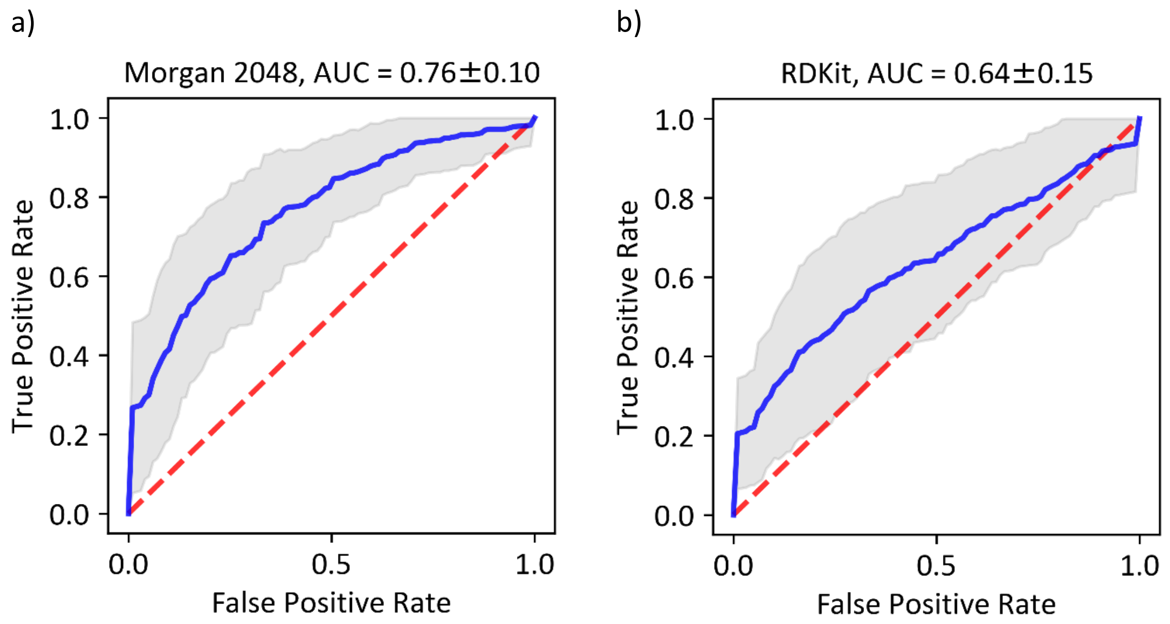

Supplementary Fig. 3: **Classification model performance using Morgan 2048 and RDKit descriptors.**

a, ROC curves for Morgan 2048-based classification models. **b,** ROC curves for RDKit descriptor-based models. 32 ROC curves (not shown for clarity) have average AUC and standard deviation of 0.78 ± 0.09. The blue line represents the average ROC curve, with the gray shaded area showing ± 1 standard deviation. The red dashed line indicates random prediction. Gamma ≤ 0.95, synergistic; Gamma > 0.95, non-synergistic. Source data are provided as a Source Data file.

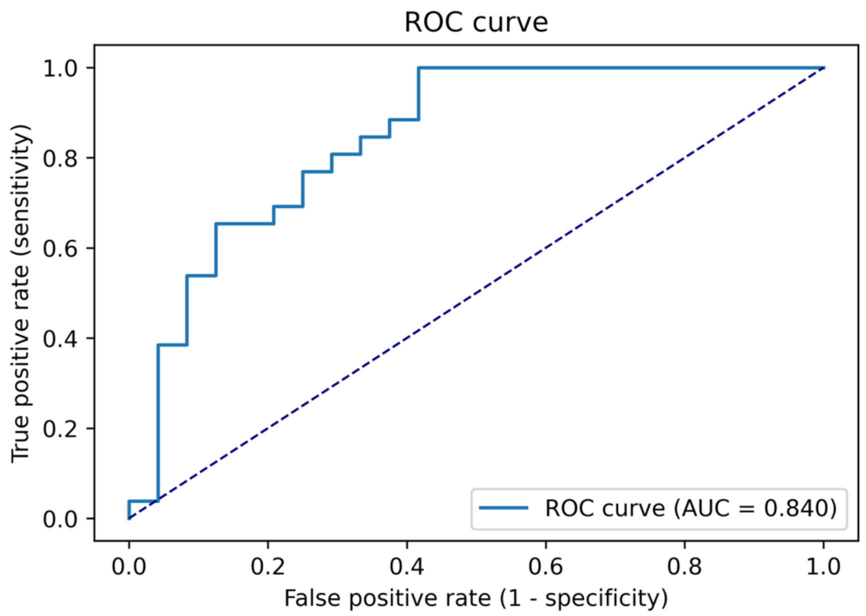

Supplementary Fig. 4: **ROC curve of the MIT graph convolutional network model.** Shown is the ROC curve for the first cross-validation fold of the graph convolutional network model developed by MIT. Gamma < 0.95 is considered synergistic, and Gamma ≥ 0.95 is non-synergistic. Source data are provided as a Source Data file.

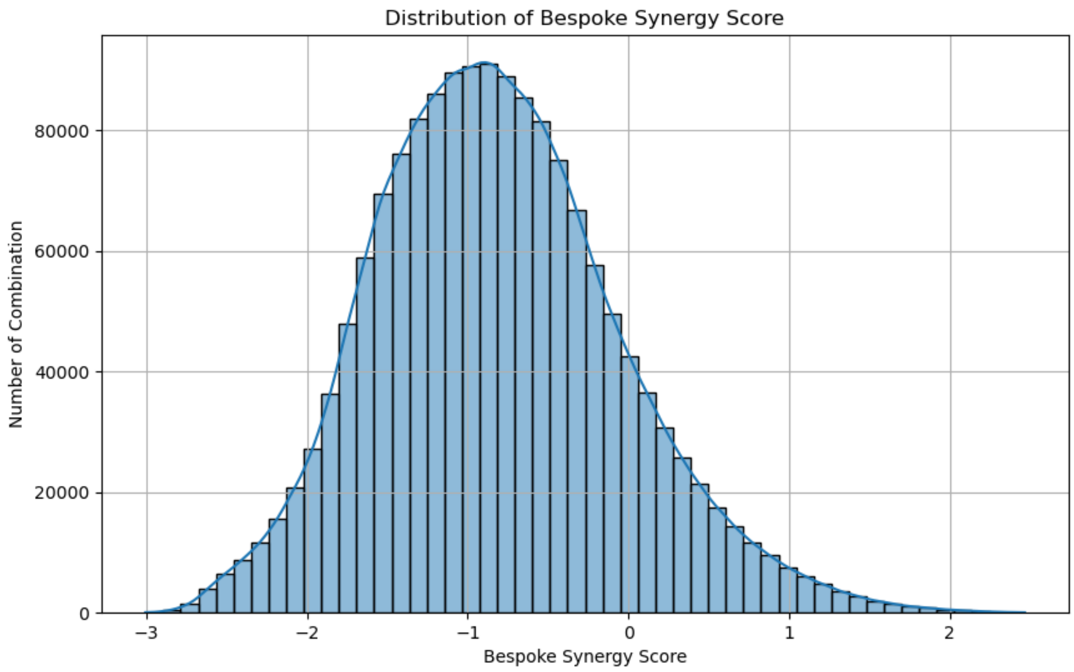

Supplementary Fig. 5: **Distribution of predicted Gamma scores across all drug combinations.** Distribution of predicted gamma scores across all 1.5M+ drug combinations evaluated by the graph convolutional model. The gamma score, a measure of synergy, is plotted on the x-axis, with

the frequency of combinations corresponding to each score shown on the y-axis. Source data are provided as a Source Data file.

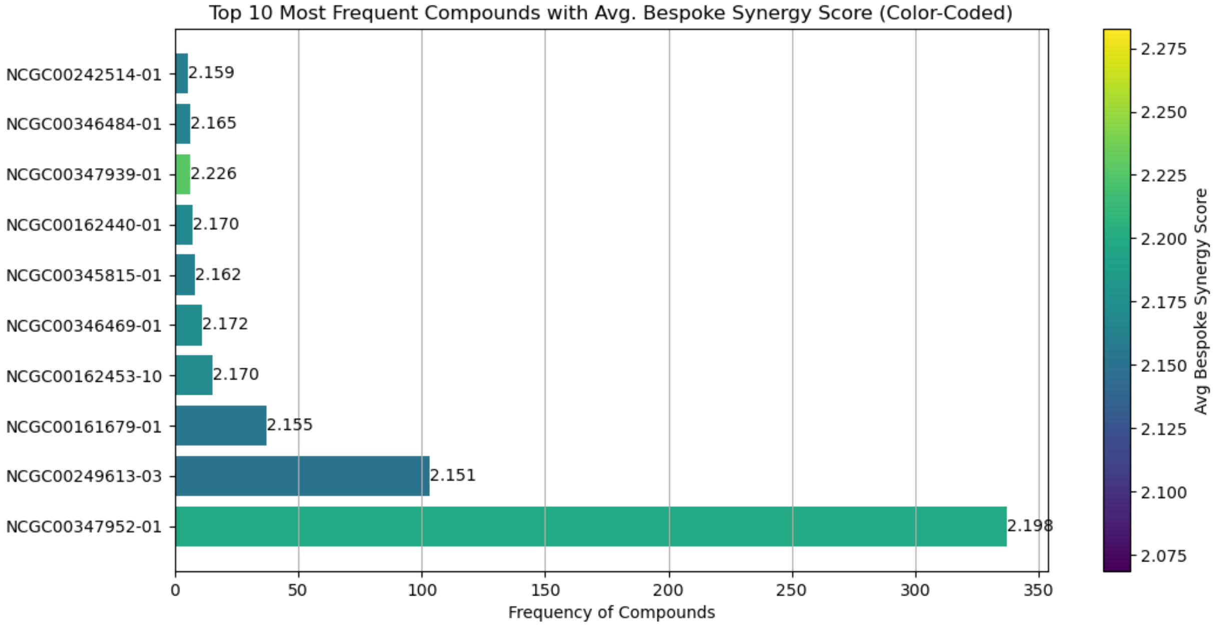

Supplementary Fig. 6: **Top compounds and their associated average Gamma scores.** Frequency and average Gamma scores of the 10 most frequently occurring compounds in synergistic combinations. Each bar represents a compound, with the length of the bar indicating the frequency of combinations involving that compound. The color gradient along the bar corresponds to the compound's average gamma score, with the color bar on the right providing a reference for interpreting these scores. Source data are provided as a Source Data file.

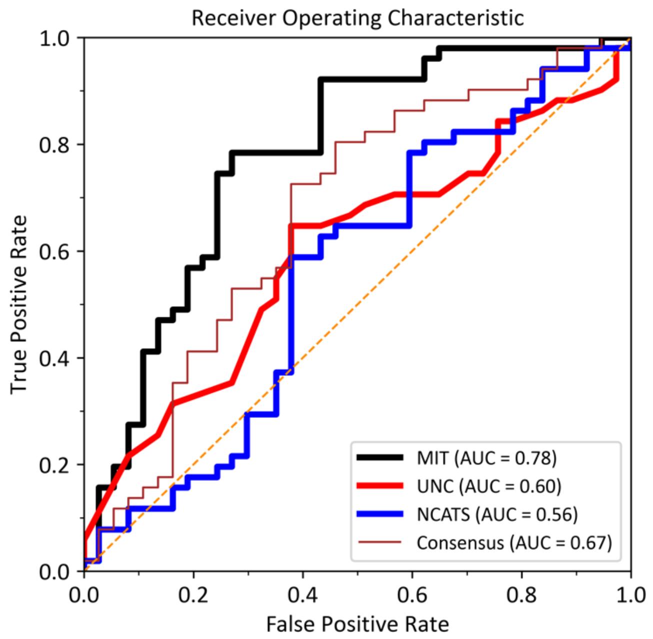

Supplementary Fig. 7: Validation of synergy predictions for experimentally tested combinations.

Retrospective evaluation of synergy predictions for 88 combinations nominated by the final models from NCATS, UNC, and MIT. Predicted synergy scores are compared to experimental results. Source data are provided as a Source Data file.

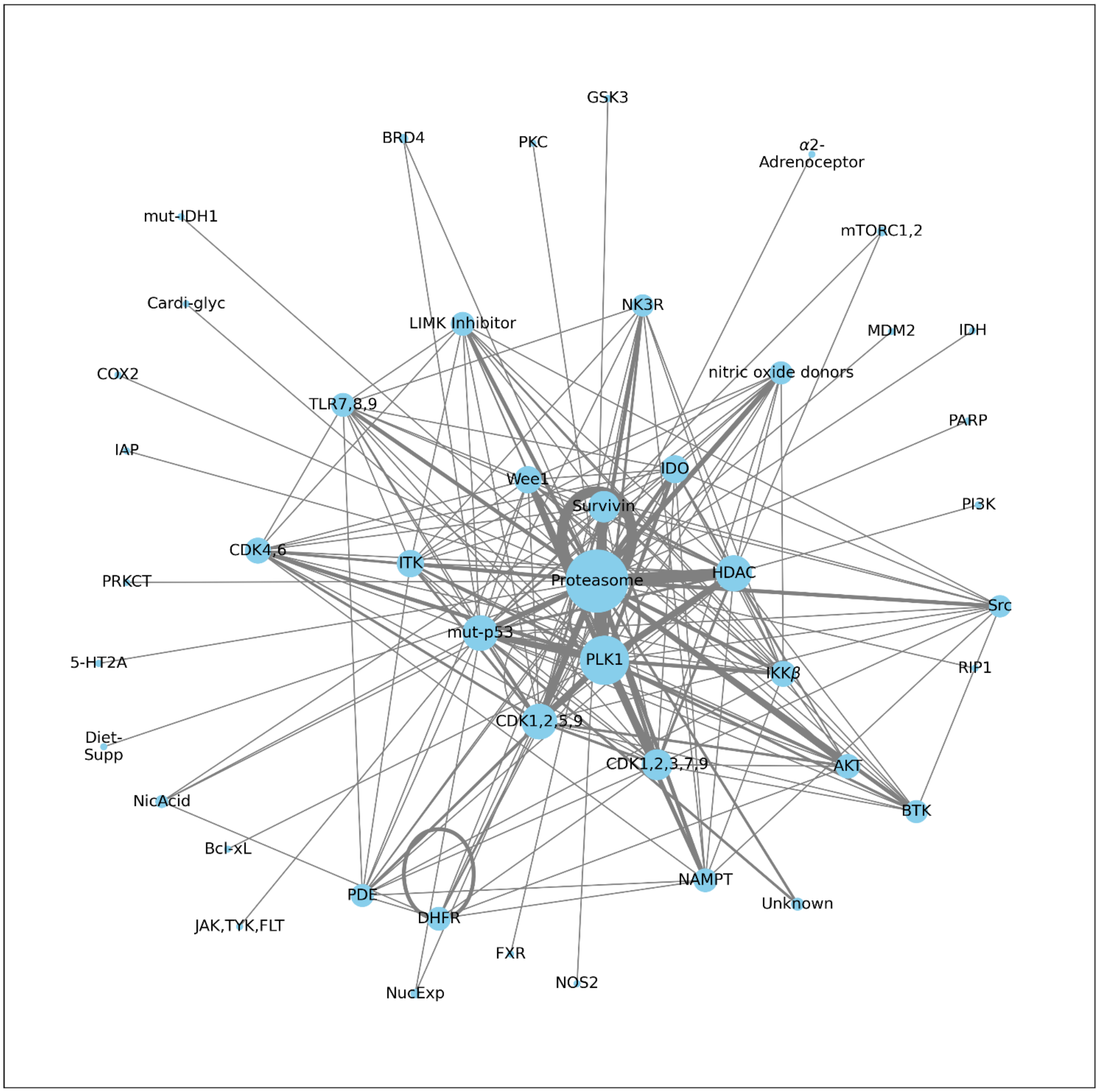

Supplementary Fig. 8: **Network analysis of mechanisms of action (MoAs) in synergistic combinations.**

The network depicts MoA interactions identified in 307 synergistic combinations (Gamma < 0.95). Node sizes and edge widths are proportional to MoA and MoA–MoA frequency, respectively. Abbreviations in alphabetical order: 5-HT2A Antagonists, 5-HT2A; AKT Inhibitor, AKT; BTK Inhibitor, BTK; Bcl-xL Inhibitor, Bcl-xL; Brd4 Inhibitor, BRD4; CDK1/2/3/7/9 Inhibitor, CDK1,2,3,7,9; CDK1/2/5/9 Inhibitor, CDK1,2,5,9; CDK4/6 Inhibitor, CDK4,6; Cardiac glycoside, Cardi-glyc; Cyclooxygenase-2 Inhibitor, COX2; Dietary supplement & part of traditional chinese medicines; alkaloid known to have several pharmacological effects including anti-tumor activity; inducer of apoptosis, Diet-Supp; Dihydrofolate Reductase (DHFR) Inhibitors, DHFR; Farnesoid X Receptor Agonist, FXR; GSK-3 Inhibitor, GSK3; HDAC Inhibitor, HDAC; IAP Inhibitor, IAP; IDO Inhibitor, IDO; IKK beta Inhibitor, IKKβ; ITK inhibitor, ITK; Isocitrate

dehydrogenase inhibitor, IDH; Jak/Tyk/Flt Inhibitor, JAK,TYK,FLT; MDM2 (hdm2) Inhibitor, MDM2; Mutant IDH1 Inhibitor, mut-IDH1; Mutant p53 Activator, mut-p53; NAMPT Inhibitor, NAMPT; NOS2 Expression Inhibitor, NOS2; Nicotinic acid, NicAcid; Nuclear export Inhibitor, NucExp; PARP Inhibitor, PARP; PI3K Inhibitor, PI3K; PKC Inhibitor, PKC; Phosphodiesterase III/Va (PDE) Inhibitor, PDE; Polo-like Kinase-1 (Plk-1) Inhibitor, PLK1; Proteasome Inhibitor, Proteasome; Protein kinase C theta Inhibitor, PRKCT; RIP1 Kinase Inhibitor, RIP1; Src Kinase Inhibitor, Src; Survivin Inhibitor, Survivin; TLR 7, 8, 9 Antagonist, TLR7,8,9; Tachykinin NK3 Antagonist, NK3R; Wee1 Kinase Inhibitor, Wee1; alpha2-Adrenoceptor Antagonist, α-Adrenoceptor; mTORC1/2 Inhibitor, mTORC1,2. Source data are provided as a Source Data file.

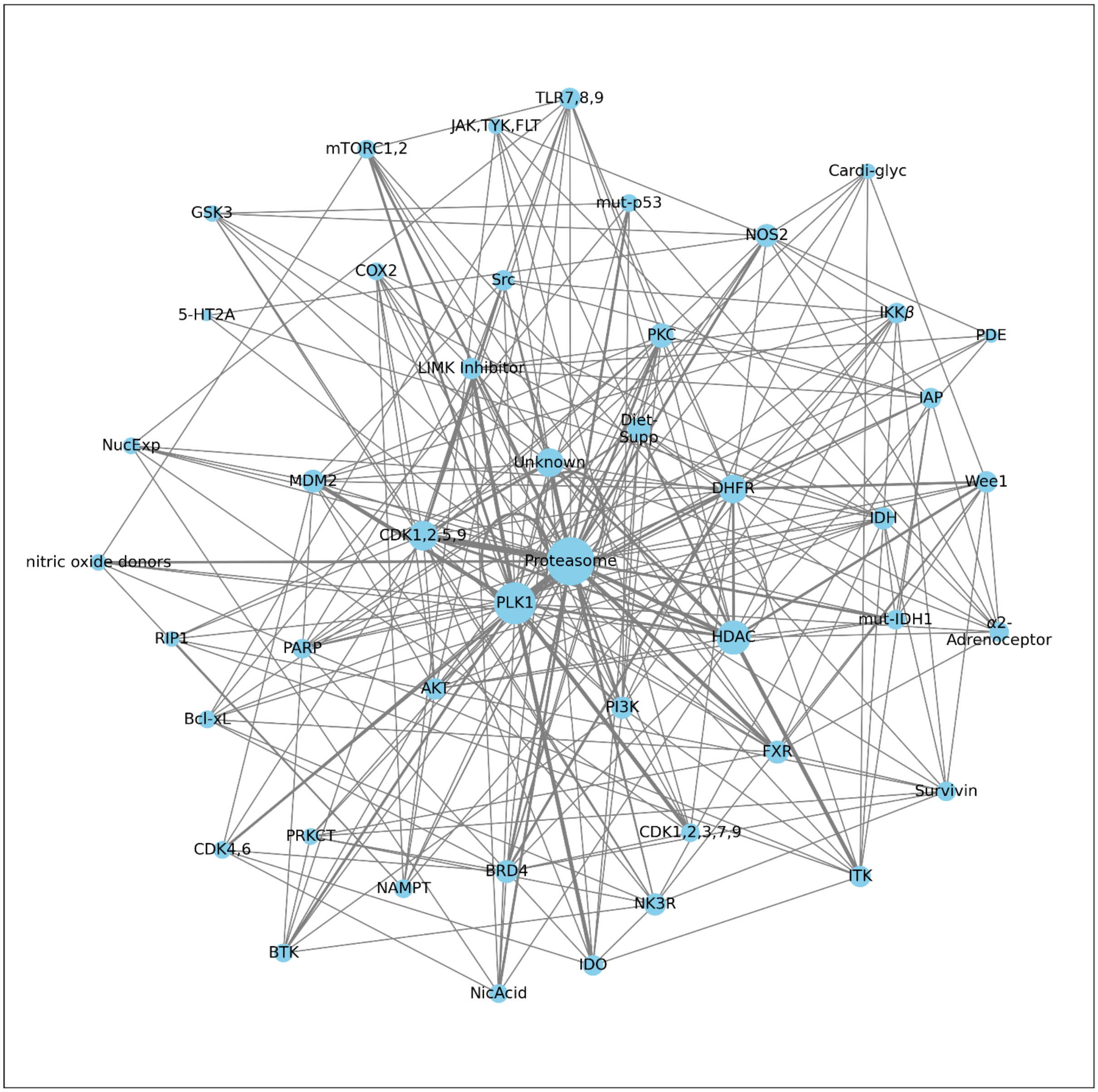

Supplementary Fig. 9: **Network analysis of MoAs in random combinations**. The analysis took the 64 unique compounds from the 307 synergistic combinations and generated 307 random combinations, repeating this 1,000 times. Node sizes and edge widths are proportional

to MoA and MoA–MoA frequency, respectively. Each MoA is abbreviated for simplicity; for example, "HDAC" refers to "HDAC Inhibitor", where HDAC is a protein name. Supplementary Fig. 8 provides details of abbreviations. Source data are provided as a Source Data file.

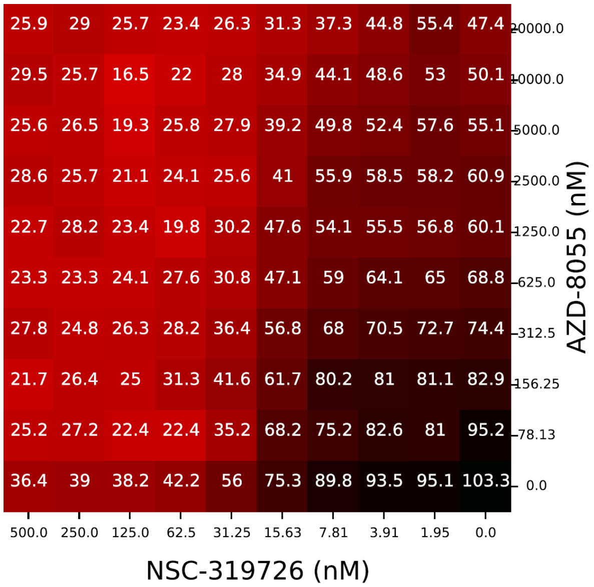

Supplementary Fig. 10: **Matrix blocks from the NSC-319726 + AZD-8055 combination assay.** Normalized activity of combinations is represented as a heat map, with 0 corresponding to full cytotoxicity and 100 to no cytotoxicity. The color gradient from black to red illustrates the progression from minimal to maximal activity, with red indicating higher activity and black representing lower activity.

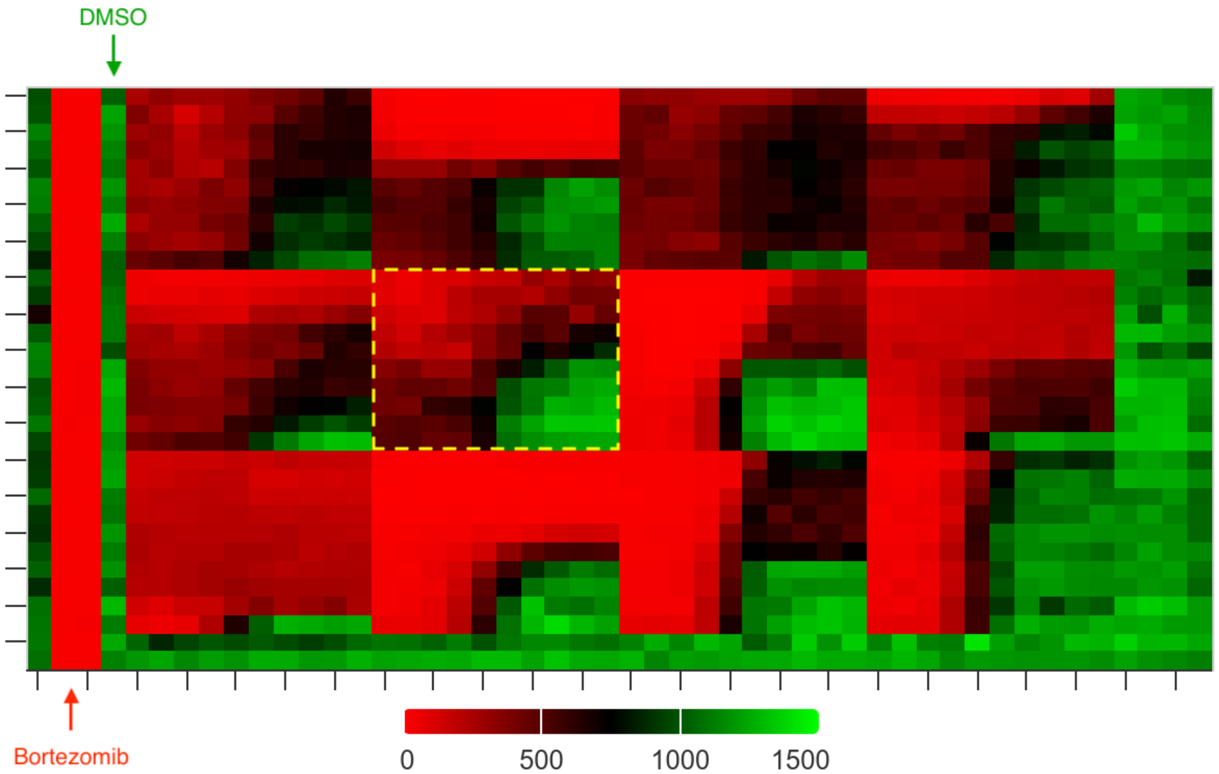

Supplementary Fig. 11: **Screening plate example with assay controls and combinations.** A screening plate example displaying responses to negative and positive controls, along with 12 combinations (highlighted by a yellow rectangle for one combination). Each 1536-well plate included DMSO as the negative control (IC0) and Bortezomib, a well-established cancer drug, as the positive control (IC100), located in the leftmost columns.

Supplementary Tables

Supplementary Table 1: Definitions of key synergy metrics in drug combination studies

Metric	Definition
**HSA**	The Highest Single Agent (HSA) model is a method used to evaluate drug synergy by comparing the effect of a drug combination to the effect of the most effective single agent within that combination. The synergy is assessed by determining whether the combined effect exceeds the maximum effect of the individual drugs when used alone.
**Excess HSA**	Excess HSA refers to the extent to which the observed effect of a drug combination exceeds the effect predicted by the Highest Single Agent (HSA) model. It quantifies the degree of synergy by measuring how much more effective the combination is compared to the most effective single agent.
**Gamma**	the Gamma (γ) score is a measure used to quantify the interaction between two or more drugs in a combination. It represents the ratio of the observed effect of the drug

	combination to the expected effect under a certain model, such as Bliss Independence or Highest Single Agent (HSA). A Gamma score helps to classify the interaction as synergistic, additive, or antagonistic [1].
**Beta**	The Beta (β) score is another metric used to assess drug interactions, particularly in the context of combinatorial drug screening. It often represents the effect size or potency of a drug combination relative to a control or reference effect. Beta scores can be used to compare the strength or efficacy of different drug combinations under various conditions [1].

1. Cokol, M. *et al.* Systematic exploration of synergistic drug pairs. *Mol. Syst. Biol.* **7**, 544 (2011).

Supplementary Table 2: Summary of NCATS machine learning results. AUC of ROC curve ± standard error of 32 values corresponding to 32 folds.

**Features**	**AUC**	**Balance Accuracy**
**Random Forest Classification**		
Avalon2048	0.78±0.02	0.68±0.01
Concatenation of Avalon1024, Morgan1024, Avalon2048, Morgan2048, RDKit, and IC50	0.77±0.02	0.69±0.02
Concatenation of Avalon2048, Morgan2048, and IC50	0.77±0.02	0.69±0.02
Morgan1024	0.77±0.02	0.68±0.02
Concatenation of Morgan2048 and Avalon2048	0.77±0.02	0.68±0.02
Majority vote of Avalon2048, Morgan2048, and RDKit	0.77±0.02	0.67±0.02
Morgan2048	0.76±0.02	0.68±0.02
Concatenation of Avalon1024, Morgan1024, and IC50	0.76±0.02	0.67±0.02
Avalon1024	0.76±0.02	0.67±0.02

Concatenation of Avalon2048 and MOA	0.76±0.02	
Average probability of Avalon2048, Morgan2048, and RDKit	0.76±0.02	0.67±0.02
Concatenation of Avalon1024 and Morgan1024	0.75±0.02	0.67±0.01
Concatenation of Morgan2048 and RDKit	0.75±0.02	0.67±0.02
Concatenation of Avalon2048 and RDKit	0.73±0.02	0.65±0.02
RDKit	0.64±0.03	0.55±0.02
**XGBoost**		
Concatenation of Avalon1024, Morgan1024, Avalon2048, Morgan2048, RDKit, and IC50	0.78±0.02	0.65±0.02
Avalon2048	0.76±0.02	0.67±0.02
Concatenation of Avalon2048, Morgan2048, and IC50	0.75±0.02	0.66±0.02
Average probability of Avalon2048, Morgan2048, and RDKit	0.75±0.02	0.65±0.02
Majority vote of Avalon2048, Morgan2048, and RDKit	0.74±0.03	0.65±0.02
Morgan2048	0.72±0.03	0.63±0.02
Concatenation of Avalon2048 and PreDx	0.7±0.03	
Concatenation of Avalon1024 and PreDx	0.68±0.03	
Concatenation of Morgan1024 and PreDx	0.68±0.03	

RDKit	0.65±0.02	0.60±0.02
**DNN**		
Avalon1024	0.76±0.02	
Morgan1024	0.76±0.02	
Morgan2048	0.75±0.02	
Concatenation of Avalon2048 and Morgan2048	0.75±0.02	
Concatenation of Avalon1024 and Morgan1024	0.75±0.02	
Concatenation of Avalon1024, Morgan1024, Avalon2048, Morgan2048, RDKit, and IC50	0.75±0.02	
Concatenation of Avalon2048, Morgan2048, and IC50	0.75±0.02	
Avalon2048	0.74±0.02	
**Averaging probabilities of random forest classification and regression**		
Avalon2048 (classification) and Avalon2048 (regression)	0.79±0.02	
Morgan2048 (classification) and Avalon2048 (regression)	0.79±0.02	
Morgan2048 (classification) and Morgan2048 (regression)	0.78±0.02	
Avalon2048 (classification) and Morgan2048 (regression)	0.78±0.02	
PreDx (classification) and Avalon2048 (regression)	0.75±0.02	
RDKit (classification) and Avalon2048 (regression)	0.74±0.02	
MOA (classification) and Avalon2048 (regression)	0.73±0.02	

PreDx (classification) and Morgan2048 (regression)	0.73±0.02	

Supplementary Table 3: UNC modeling results

One-Compound Out, not using IC50

Descriptor	Descriptor Composition Strategy	Model Name	**CCR**	**PPV**	NPV	SE	SP	Number of samples/tests	Test Size	Train Size
RDKit Descriptors	Average	Gradient Boosting	**0.59**	**0.60**	0.59	0.63	0.56	20	256	120
RDKit Descriptors	Average	Neural Network	**0.61**	**0.64**	0.58	0.54	0.67	20	256	120
RDKit Descriptors	Average	Random Forest	**0.64**	**0.66**	0.62	0.66	0.62	20	256	120
RDKit Descriptors	Element-wise sum	Gradient Boosting	**0.62**	**0.63**	0.60	0.61	0.63	20	256	120
RDKit Descriptors	Element-wise sum	Neural Network	**0.57**	**0.60**	0.55	0.42	0.71	20	256	120
RDKit Descriptors	Element-wise sum	Random Forest	**0.62**	**0.65**	0.60	0.63	0.61	20	256	120
Simplex Descriptor	N/A	Gradient Boosting	**0.59**	**0.59**	0.59	0.62	0.55	20	256	120
Simplex Descriptor	N/A	Neural Network	**0.61**	**0.63**	0.59	0.57	0.65	20	256	120
Simplex Descriptor	N/A	Random Forest	**0.64**	**0.64**	0.63	0.68	0.59	20	256	120
Morgan Fingerprint	Average	Gradient Boosting	**0.65**	**0.65**	0.64	0.67	0.63	20	256	120
Morgan Fingerprint	Average	Neural Network	**0.61**	**0.65**	0.59	0.52	0.71	20	256	120
Morgan Fingerprint	Average	Random Forest	**0.65**	**0.65**	0.65	0.67	0.63	20	256	120
Morgan Fingerprint	Element-wise sum	Gradient Boosting	**0.63**	**0.63**	0.62	0.66	0.59	20	256	120
Morgan Fingerprint	Element-wise sum	Neural Network	**0.60**	**0.68**	0.55	0.41	0.79	20	256	120
Morgan Fingerprint	Element-wise sum	Random Forest	**0.66**	**0.66**	0.66	0.68	0.64	20	256	120

One-Compound-Out, using IC50

Descriptor	Descriptor Composition Strategy	Model Name	**CCR**	**PPV**	NPV	SE	SP	Number of samples/tests	Test Size	Train Size
Molecular Graph + IC50	Concatenate	Graph Convolution	**0.64**	**0.65**	0.63	0.63	0.63	20	256	120
Simplex + IC50	Concatenate	Simplex Neural Network	**0.64**	**0.65**	0.62	0.61	0.65	20	256	120

Everything-Out, not using IC50

Descriptor	Descriptor Composition Strategy	Model Name	**CCR**	**PPV**	NPV	SE	SP	Number of samples/tests	Train Size	Test Size
Morgan Fingerprint	Average	Gradient Boosting	**0.59**	**0.61**	0.58	0.62	0.57	20	120	120
Morgan Fingerprint	Average	Neural Network	**0.52**	**0.55**	0.50	0.27	0.77	20	120	120
Morgan Fingerprint	Average	Random Forest	**0.53**	**0.55**	0.52	0.65	0.42	20	120	120
Morgan Fingerprint	Element-wise sum	Gradient Boosting	**0.54**	**0.55**	0.54	0.64	0.45	20	120	120
Morgan Fingerprint	Element-wise sum	Neural Network	**0.52**	**0.55**	0.49	0.42	0.63	20	120	120
Morgan Fingerprint	Element-wise sum	Random Forest	**0.53**	**0.55**	0.51	0.55	0.51	20	120	120
RDKit Descriptors	Average	Gradient Boosting	**0.53**	**0.55**	0.51	0.53	0.53	20	120	120
RDKit Descriptors	Average	Neural Network	**0.50**	**0.51**	0.48	0.38	0.61	20	120	120
RDKit Descriptors	Average	Random Forest	**0.54**	**0.55**	0.52	0.54	0.53	20	120	120
RDKit Descriptors	Element-wise sum	Gradient Boosting	**0.52**	**0.54**	0.49	0.50	0.54	20	120	120
RDKit Descriptors	Element-wise sum	Neural Network	**0.49**	**0.50**	0.48	0.32	0.66	20	120	120
RDKit Descriptors	Element-wise sum	Random Forest	**0.54**	**0.55**	0.53	0.61	0.47	20	120	120
Simplex Descriptor	N/A	Gradient Boosting	**0.50**	**0.53**	0.47	0.49	0.51	20	120	120
Simplex Descriptor	N/A	Neural Network	**0.46**	**0.48**	0.44	0.43	0.49	20	120	120
Simplex Descriptor	N/A	Random Forest	**0.50**	**0.52**	0.49	0.52	0.48	20	120	120

Everything-Out, using IC50

Descriptor	Descriptor Composition Strategy	Model Name	**CCR**	**PPV**	NPV	SE	SP	Number of samples/tests	Test Size	Train Size
Molecular Graph + IC50	Concatenate	Graph Convolution	**0.56**	**0.57**	0.54	0.58	0.53	20	120	120
Simplex Descriptor + IC50	Concatenate	Neural Network	**0.53**	**0.56**	0.51	0.51	0.56	20	120	120

Supplementary Table 4: Cross-validation accuracy of the MIT graph convolutional network model on each of the five cross validation folds.

Cross validation fold	AUROC
1	0.840
2	0.771
3	0.849
4	0.870
5	0.868

Supplementary Table 5: List of 26 strongly synergistic combinations with gamma < 0.5.

**Gamma**	**Compound 1**	**Compound 2**	**MoA 1**	**MoA 2**
**0.041**	Panobinostat	Carfilzomib	HDAC Inhibitor	Proteasome Inhibitor
**0.108**	Oprozomib	Panobinostat	Proteasome Inhibitor	HDAC Inhibitor
**0.224**	Trichostatin A	Sepantronium bromide	HDAC Inhibitor	Survivin Inhibitor
**0.240**	Trichostatin A	Carfilzomib	HDAC Inhibitor	Proteasome Inhibitor
**0.260**	Quisinostat	Carfilzomib	HDAC Inhibitor	Proteasome Inhibitor
**0.299**	NSC-319726	Panobinostat	Mutant p53 Activator	HDAC Inhibitor
**0.305**	NCGC00262689	Carfilzomib	Isocitrate dehydrogenase inhibitor	Proteasome Inhibitor
**0.305**	Carfilzomib	NCGC00188382-01	Proteasome Inhibitor	ITK inhibitor

**0.308**	AGI-5198	Carfilzomib	Mutant IDH1 Inhibitor	Proteasome Inhibitor
**0.335**	Sotrastaurin	Carfilzomib	Protein kinase C theta Inhibitor	Proteasome Inhibitor
**0.346**	Go-6983	Carfilzomib	PKC Inhibitor	Proteasome Inhibitor
**0.355**	Panobinostat	Bortezomib	HDAC Inhibitor	Proteasome Inhibitor
**0.362**	Ketanserin	Carfilzomib	5-HT2A Antagonists	Proteasome Inhibitor
**0.363**	Carfilzomib	Daporinad	Proteasome Inhibitor	NAMPT Inhibitor
**0.366**	Yohimbine	Carfilzomib	alpha2-Adrenoceptor Antagonist	Proteasome Inhibitor
**0.367**	GMX-1778	Carfilzomib	IKK beta Inhibitor	Proteasome Inhibitor
**0.378**	Dacinostat	Carfilzomib	HDAC Inhibitor	Proteasome Inhibitor
**0.380**	Carfilzomib	Bortezomib	Proteasome Inhibitor	Proteasome Inhibitor
**0.399**	SB-216763	Carfilzomib	GSK-3 Inhibitor	Proteasome Inhibitor
**0.402**	Turofexorate isopropyl	Carfilzomib	Farnesoid X Receptor Agonist	Proteasome Inhibitor
**0.406**	Carfilzomib	GW-843682X	Proteasome Inhibitor	Polo-like Kinase-1 (Plk-1) Inhibitor
**0.409**	Melatonin	Carfilzomib	NOS2 Expression Inhibitor	Proteasome Inhibitor
**0.417**	Necrostatin-1	Carfilzomib	RIP1 Kinase Inhibitor	Proteasome Inhibitor
**0.420**	Rutaecarpine	Carfilzomib	Cyclooxygenase-2 Inhibitor	Proteasome Inhibitor
**0.475**	Evodiamine	Carfilzomib	Dietary supplement & part of traditional chinese medicines; alkaloid known to have several pharmacological effects including anti-tumor activity; inducer of apoptosis	Proteasome Inhibitor
**0.498**	Oprozomib	Carfilzomib	Proteasome Inhibitor	Proteasome Inhibitor

Supplementary Table 6. Small molecule screening data

Category	Parameter	Description

Assay	Type of assay	Quantitative high-throughput screening (qHTS) for single-agent and combination drug responses in PANC-1 cells.
	Target	PANC-1 pancreatic cancer cells (human carcinoma of the exocrine pancreas).
	Primary measurement	IC50 values and cell viability (using ATP-based detection with CellTiter-Glo® Luminescent Cell Viability Assay).
	Key reagents	CellTiter-Glo® Luminescent Cell Viability Assay, PANC-1 cell line, DMSO, Bortezomib as a positive control.
	Assay protocol	PANC-1 cells (500 cells/5 µL/well) seeded in 1536-well plates. Cells stimulated with compounds for 72 hours at 37°C, 5% CO_2. Cell viability measured using CellTiter-Glo® after 72 hours.
	Additional comments	The assay was run in duplicate to ensure robustness. Positive control: Bortezomib; negative control: DMSO.
Library	Library size	~2,000 compounds (MIPE4 library)
	Library composition	Antineoplastic compounds with diverse and redundant mechanisms of action, including approved drugs, investigational drugs (phases I−III), and pre-clinical molecules.
	Source	NCATS in-house MIPE4 library
	Additional comments	The library consists of compounds with varied mechanisms of action, aiming to identify potential synergies in pancreatic cancer treatment.
Screen	Format	1536-well plate format
	Concentration(s) tested	11 serial dilutions ranging from 46 µM to 0.78 nM (single-agent dose-response format).
	Plate controls	Positive control: Bortezomib (IC100); Negative control: DMSO (IC0)
	Reagent/ compound dispensing system	MultiDrop Combi dispenser (Thermo Scientific), Pintool transfer (Kalypsis), Echo 650 acoustic liquid handler (Beckman Coulter).

	Detection instrument and software	ViewLux plate reader (PerkinElmer) with 3-second exposure time for luminescence detection.
	Assay validation/QC	Z' factor > 0.68; Assays were performed in duplicate, using both positive and negative controls (Bortezomib and DMSO, respectively). Plates were processed consistently to ensure robust and reliable data.
	Correction factors	N/A
	Normalization	Percent inhibition compared to positive (Bortezomib) and negative (DMSO) controls.
	Additional comments	CellTiter-Glo® luminescent assay used for reliable ATP-based detection of viable cells.
Post-HTS analysis	Hit criteria	IC50 values for compounds showing at least 50% efficacy and following curve classes 1.1 and 1.2.
	Hit rate	32 compounds identified with IC50 values and efficacy above 50%.
	Additional assay(s)	Matrix screening for 496 combinations (10x10 matrices) using nine 1:2 serial dilutions. Each combination was tested in duplicates.
	Confirmation of hit purity and structure	Re-testing of the 32 identified compounds for validation of IC50 and efficacy in combination screening.
	Additional comments	Compounds pre-dispensed to plates, followed by addition of PANC-1 cells and CellTiter-Glo® reagent for final detection. Screening results validated using curve classes and matrix assays.
