# Supplementary material for: AI-driven discovery of synergistic drug combinations against pancreatic cancer
Source: Nat Commun. 2025 Apr 29;16:4020. doi: 10.1038/s41467-025-56818-6 (PMC12041571; doi:10.1038/s41467-025-56818-6)
Supplement: Supplementary file 2 — Reporting Summary [file 41467_2025_56818_MOESM2_ESM.pdf]

nature portfolio

Corresponding author(s): Alexey V. Zakharov

Last updated by author(s): Nov 27, 2024

Reporting Summary

Nature Portfolio wishes to improve the reproducibility of the work that we publish. This form provides structure for consistency and transparency in reporting. For further information on Nature Portfolio policies, see our Editorial Policies and the Editorial Policy Checklist.

Statistics

For all statistical analyses, confirm that the following items are present in the figure legend, table legend, main text, or Methods section.

n/a	Confirmed	
☐	☒	The exact sample size (n) for each experimental group/condition, given as a discrete number and unit of measurement
☒	☐	A statement on whether measurements were taken from distinct samples or whether the same sample was measured repeatedly
☒	☐	The statistical test(s) used AND whether they are one- or two-sided<br>*Only common tests should be described solely by name; describe more complex techniques in the Methods section.*
☒	☐	A description of all covariates tested
☒	☐	A description of any assumptions or corrections, such as tests of normality and adjustment for multiple comparisons
☐	☒	A full description of the statistical parameters including central tendency (e.g. means) or other basic estimates (e.g. regression coefficient) AND variation (e.g. standard deviation) or associated estimates of uncertainty (e.g. confidence intervals)
☒	☐	For null hypothesis testing, the test statistic (e.g. F, t, r) with confidence intervals, effect sizes, degrees of freedom and P value noted<br>*Give P values as exact values whenever suitable.*
☒	☐	For Bayesian analysis, information on the choice of priors and Markov chain Monte Carlo settings
☒	☐	For hierarchical and complex designs, identification of the appropriate level for tests and full reporting of outcomes
☐	☒	Estimates of effect sizes (e.g. Cohen's d, Pearson's r), indicating how they were calculated

Our web collection on statistics for biologists contains articles on many of the points above.

Software and code

Policy information about availability of computer code

Data collection	For experimental data, high-throughput screening assays were conducted using the MIPE4 compound library on PANC-1 pancreatic cancer cell lines, with data analysis performed via the internal QHTS client software. In silico data were generated using custom machine learning models based on molecular descriptors and biological data to predict drug combination synergy. Fingerprints (Avalon1024, Avalon2048, Morgan1024, Morgan2048) and RDKit descriptors were generated using the RDKit package (https://rdkit.org), while biological descriptors were obtained using NCATS Predictor (https://predictor.ncats.io/) via in-house Python 3.7.7 scripts. All data were collected following established protocols to ensure consistency and accuracy.
Data analysis	Data processing, model training, and validation were performed using custom Python 3.7.7 and R scripts. All analysis workflows are available upon request, and the code has been made available in a community repository for transparency and reproducibility.

For manuscripts utilizing custom algorithms or software that are central to the research but not yet described in published literature, software must be made available to editors and reviewers. We strongly encourage code deposition in a community repository (e.g. GitHub). See the Nature Portfolio guidelines for submitting code & software for further information.

Data

Policy information about availability of data

All manuscripts must include a data availability statement. This statement should provide the following information, where applicable:

- Accession codes, unique identifiers, or web links for publicly available datasets
- A description of any restrictions on data availability
- For clinical datasets or third party data, please ensure that the statement adheres to our policy

All data generated or analyzed during this study are included in this published article and its supplementary information files. Custom scripts and models used for the analysis have been deposited in a public GitHub repository (https://github.com/ncats/PANC1), ensuring transparency and reproducibility.

Research involving human participants, their data, or biological material

Policy information about studies with human participants or human data. See also policy information about sex, gender (identity/presentation), and sexual orientation and race, ethnicity and racism.

Reporting on sex and gender	N/A
Reporting on race, ethnicity, or other socially relevant groupings	N/A
Population characteristics	N/A
Recruitment	N/A
Ethics oversight	N/A

Note that full information on the approval of the study protocol must also be provided in the manuscript.

Field-specific reporting

Please select the one below that is the best fit for your research. If you are not sure, read the appropriate sections before making your selection.

☒ Life sciences ☐ Behavioural & social sciences ☐ Ecological, evolutionary & environmental sciences

For a reference copy of the document with all sections, see nature.com/documents/nr-reporting-summary-flat.pdf

Life sciences study design

All studies must disclose on these points even when the disclosure is negative.

Sample size	The sample size was determined based on the standard high-throughput screening protocols and the availability of compounds in the MIPE4 library.
Data exclusions	No data were excluded from the analysis.
Replication	The assay was run in duplicate to ensure robustness. Bortezomib was used as the positive control and DMSO as the negative control.
Randomization	Randomization was not applicable as this study involved predefined compound libraries.
Blinding	Blinding was not applicable, as the study focused on in vitro screening and in silico modeling.

Reporting for specific materials, systems and methods

We require information from authors about some types of materials, experimental systems and methods used in many studies. Here, indicate whether each material, system or method listed is relevant to your study. If you are not sure if a list item applies to your research, read the appropriate section before selecting a response.

Materials & experimental systems

n/a	Involved in the study
☒	☐ Antibodies
☐	☒ Eukaryotic cell lines
☒	☐ Palaeontology and archaeology
☒	☐ Animals and other organisms
☒	☐ Clinical data
☒	☐ Dual use research of concern
☒	☐ Plants

Methods

n/a	Involved in the study
☒	☐ ChIP-seq
☒	☐ Flow cytometry
☒	☐ MRI-based neuroimaging

Eukaryotic cell lines

Policy information about cell lines and Sex and Gender in Research

Cell line source(s)	PANC-1 pancreatic cancer cells (human carcinoma of the exocrine pancreas)
Authentication	all cells were regularly tested through authentication procedure with outsourced STR testing.
Mycoplasma contamination	the cell line was tested negative for mycoplasma prior to all experiments
Commonly misidentified lines (See ICLAC register)	N/A

Plants

Seed stocks	N/A
Novel plant genotypes	N/A
Authentication	N/A
